# CT in ovarian cancer staging: how to review and report with emphasis on abdominal and pelvic disease for surgical planning

**DOI:** 10.1186/s40644-016-0076-2

**Published:** 2016-08-02

**Authors:** Anju Sahdev

**Affiliations:** St Bartholomew’s Hospital, Barts Health, West Smithfield, London, EC1A 7BE UK

**Keywords:** CT, Ovarian cancer, Disease distribution, Template reporting, Surgical resection

## Abstract

CT of the abdomen and pelvis is the first line imaging modality for staging, selecting treatment options and assessing disease response in ovarian cancer. The staging CT provides disease distribution, disease burden and is the imaging surrogate for surgico-pathological FIGO staging. Optimal cyto-reductive surgery offers patients’ the best chance for disease control or cure, but sub-optimal resection confers no advantage over chemotherapy and adversely increases the risk of post surgical complications. Although there is extensive literature comparing performance of CT against laparoscopy and surgery, for the staging abdominal and pelvic CT, there are currently no accepted guidelines for interpretation or routinely used minimum data set templates for reporting these complex CT scans often with extensive radiological findings. This review provides a systematic approach for identifying the important radiological findings and highlighting important sites of disease within the abdomen and pelvis, which may alter or preclude surgery at presentation or after adjuvant chemotherapy. The distribution of sites and volume of disease can be used to categorize patients as suitable, probably suitable or not suitable for optimal cyto-reductive surgery. This categorization can potentially assist oncological surgeons and oncologists as a semi objective assessment tool useful for selecting patient treatment, streamlining multi disciplinary discussion and improving the reproducibility and correlation of CT with surgical findings. The review also highlights sites of disease and complications of ovarian cancer which should be included as part of the radiological report as these may require additional surgical input from non gynaecological surgeons or influence treatment selection.

## Background

CT of the abdomen and pelvis is the standard imaging modality for preoperative imaging staging at presentation and in distinguishing between patients suitable for primary cyto-reductive surgery and patients requiring neoadjuvant chemotherapy prior to surgery. Abdominal and pelvic peritoneal disease is present in more than 70 % of the women at the time of presentation. The optimal standard of care for patients with ovarian cancer, is either primary cyto-reductive surgery or adjuvant platinum based chemotherapy followed by cyto-reductive surgery [1].

CT has been validated as an accurate imaging method to predict successful surgical cyto-reduction [2, 3]. The extent and distribution of disease on CT determines whether complete cyto-reduction can be performed. Although there is no clear consensus on the criteria for resectability, when CT features are favourable, primary cyto-reductive surgery with complete resection (R0) or residual disease less than 1 cm (R1) resection offers the patient best chance for cure. Neoadjuvant chemotherapy with interval cyto-reductive surgery after 3 cycles of chemotherapy may be of benefit in selected patients when up front primary cyto-reductive surgery is unlikely to yield less than 1 cm residual abdominal-pelvic disease. During primary surgery for advanced stage epithelial ovarian cancer all attempts should be made to achieve complete cyto-reduction. When this is not achievable, the surgical goal should be optimal (<1 cm) residual disease [1, 4].

When primary chemotherapy is offered, an imaging guided biopsy, planned from the staging CT, confirms diagnosis and provides histological sub-type. Increasingly, CT of the chest is included for detecting para-cardiac nodes, pleural and pulmonary metastases. Preoperative detection of enlarged para-cardiac nodes and moderate or large pleural effusion independently predicts poor post-treatment outcome [5, 6].

This article aims to review CT features for staging ovarian cancer, highlighting routes of dissemination, important features for surgical planning and guiding cyto-reductive surgery. A systematic method of evaluating the CT and presenting the features is suggested.

## Review

### CT Technique

The optimal abdominal and pelvic CT technique for staging ovarian cancer includes oral contrast media to opacify small bowel and in the absence of contraindications to intravenous contrast media, the use of intravenous contrast media is mandatory. Oral contrast media helps to distinguish between luminal lesions and serosal or mesenteric deposits. However care should be taken as small, calcified deposits may be masked by oral contrast. Due to this reason, institutions may choose to use oral water as negative contrast.

Image acquisition should be performed 70 s after injection of contrast media. Images should be reconstructed with an axial slice thickness of 1-2 mm, coronal and sagittal slice thickness of 3-5 mm. Acquisition for an abdominal and pelvic CT should extend from the distal chest to the inguinal regions [7].

In our institute the whole chest is routinely included to detect pleural deposits, mediastinal and hilar lymph nodes and lung metastases, which can occur in up to 30 % of patients at presentation.

### Limitations

The most important limitation of CT is its inability to demonstrate small volume extra-ovarian <5 mm deposits on bowel serosa, mesentery and peritoneum especially in the absence of ascites, necessitating the complementary role of laparoscopy in pre surgical evaluation of ovarian cancer. The sub diaphragmatic regions were historically a challenging area for CT, but with multi-planar reformatting now widely available, this limitation can be overcome. Quantifying the extent of mesenteric and serosal disease is both technically difficult to achieve and difficult to communicate in a written report. Despite these limitations, with good acquisition and reading, its performance remains excellent with reported accuracies of 70-90 % for detection of disease at all stages [8]. Inability to use intravenous contrast media adversely affects CT image quality and performance.

### Routes of dissemination

The most frequent routes for dissemination are by direct pelvic invasion and via transcoelomic peritoneal disease extension. Direct invasion in the pelvis involves the adnexa, uterus, bladder, rectum and pelvic side walls. Transcoelomic spread involves the peritoneal, subdiaphragmatic mesenteric and serosal surfaces. Lymphatic metastases occur to pelvic, abdominal, retroperitoneal, para-aortic and mediastinal nodes. Haematogenous metastases are least common and most frequently occur in the liver, lung, bone and brain.

#### A: Abdominal and pelvic disease evaluation

##### Key Sites of disease for surgical planning

**Fig. 1:**
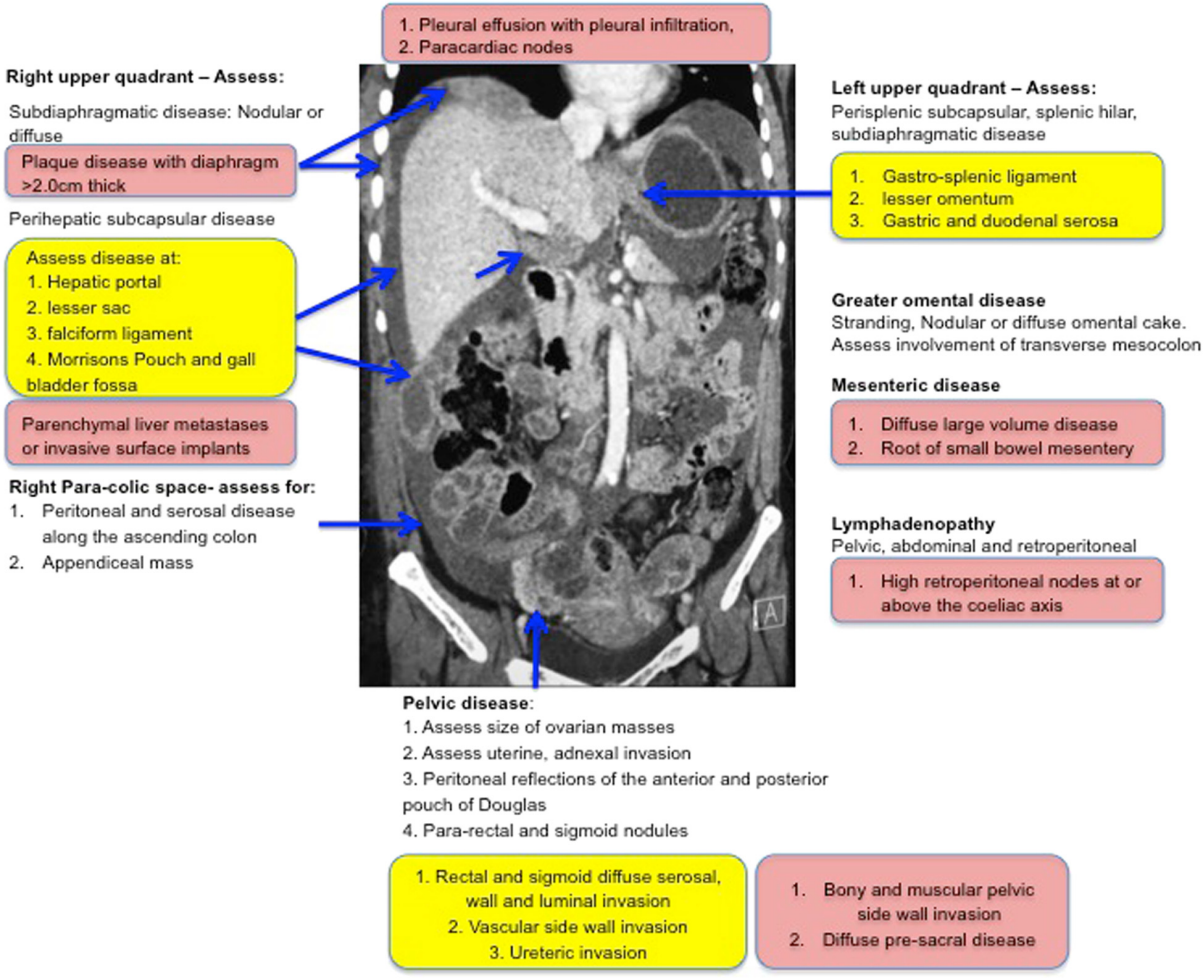
Schematic of the common sites of disease in ovarian cancer. The features in yellow are sites of potentially non resectable disease. The features in red are sites of non resectable disease

Figure 1 summarises the frequent sites of disease within the abdomen and pelvis. It provides a schematic approach describing the disease expected at each site. Each site can be approached systematically, starting from the pelvis, into the right hemi-abdomen, then the upper abdominal ligaments, sub diaphragmatic distribution, left hemi-abdomen, central abdomen and solid viscera. Features highlighted in the yellow boxes represent potentially non-resectable disease, which may preclude optimal cyto-reductive surgery. In centres with an aggressive surgical approach, these sites of disease may be deemed resectable with input from the appropriate specialty surgeons. The features highlighted in the red boxes are at present considered non-resectable disease as resection is unlikely to achieve optimal cyto-reduction. Extensive surgical resections are always possible but confer an increased surgical morbidity and mortality. At present there are no clear established surgical criteria for inoperable disease or even a universally accepted definition for optimal cyto-reduction. The working rules are based on clinical trials demonstrating a good prognosis and long-term survival when less than 1 cm of residual tumour is present at completion of cyto-reductive surgery [1, 4, 8, 9].

**Pelvic disease:**

**Fig. 2:**
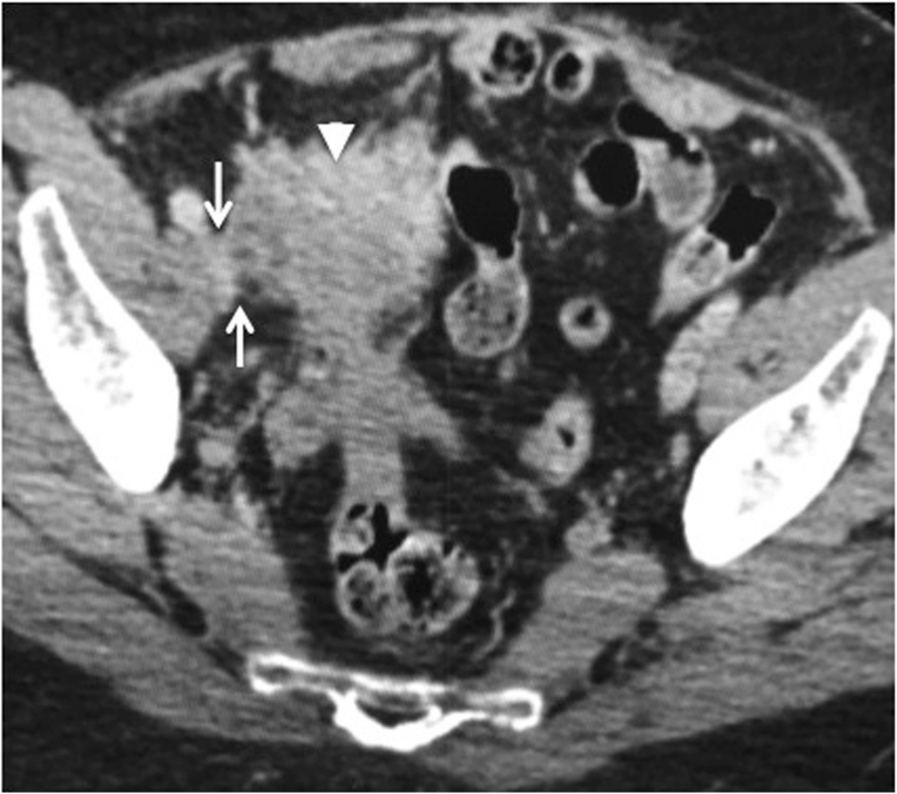
Seventy-six year old woman with a right sided malignant ovarian mass (arrow head). The mass extends to the right pelvic side wall and abuts the right external iliac vein (arrows). A distance of less than 3 mm from the pelvic side wall structures is highly suggestive of invasion

To guide pelvic surgery, the following should be included in the radiologists report:

- The size, location and extent of primary ovarian carcinoma in the pelvis.
- The presence of bladder, ureteric and rectal invasion. This will require surgical modification to include ureteric stenting, partial or complete cystectomy and bowel stoma.
- The presence of pelvic sidewall invasion, which should be suspected if the disease is less than 3 mm from the muscular sidewall and there is invasion or encasement of the iliac vessels. These may preclude surgical cyto-reductive [Fig. 2].

**Peritoneal disease:**

**Fig. 3:**
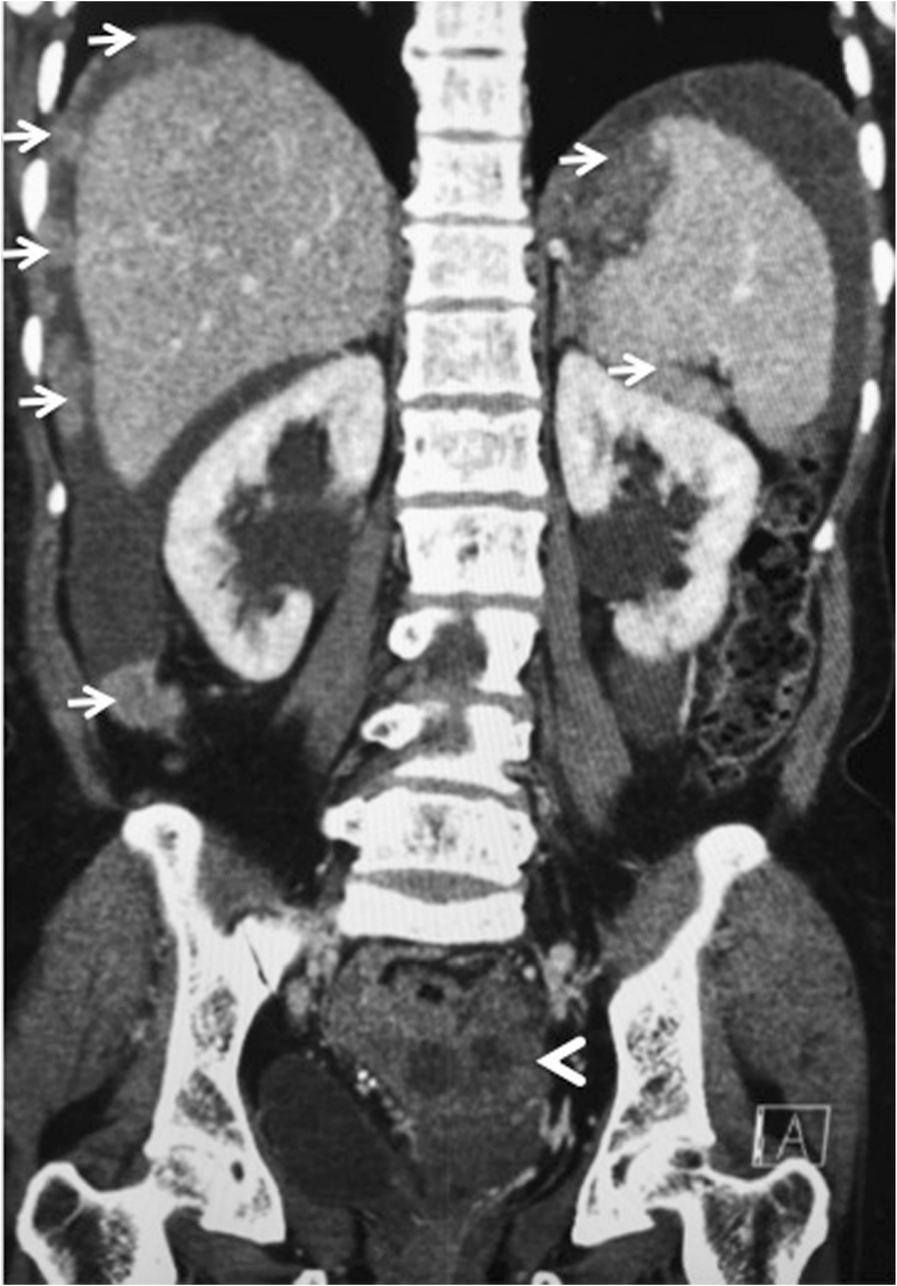
Eighty year old woman with stage IIIC papillary serous adenocarcinoma of the ovary (arrowhead). Extensive resectable metastatic peritoneal nodules are arrowed in the abdomen. The pelvic disease causes bilateral ureteric obstruction with resultant bilateral hydronephrosis

The subtle signs of peritoneal disease can easily be missed. Abnormal enhancement of the peritoneum, subtle thickening and fine reticular nodular pattern along the peritoneal surface are the early signs. The most common sites are along the pelvic peritoneal reflection, the right paracolic gutter along the caecum and ascending colon and left para colic gutter. Peritoneal deposits maybe calcified particularly in serous cystadenocarcinomas. Advanced stage peritoneal disease most frequently presents as large nodular deposits [Fig. 3].

**Subdiaphragmatic disease:**

This is best evaluated on a combination of axial and coronal CT. The right is more commonly involved due to preferential transcoelomic flow in the abdominal cavity. Similar to peritoneal and omental disease, early signs include abnormal hepatic capsular or diaphragmatic enhancement progressing to nodular disease and dense plaque disease. There is wide institutional variation on the degree of diaphragmatic disease that is considered resectable. Nontheless, most early low volume disease can be stripped particularly when assisted by newer plasma surgery systems for tissue coagulation (eg Plasma jet).

**Fig. 4:**
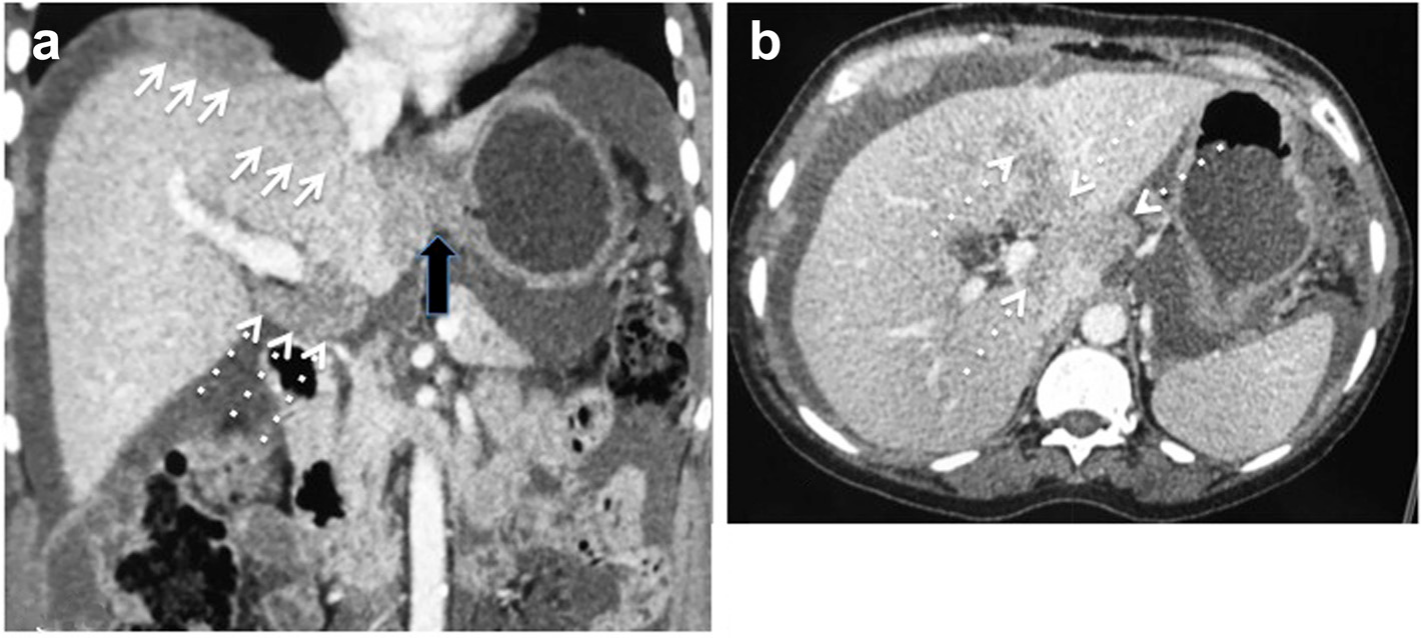
**a** and **b** 64 year old woman with stage IIIC clear cell carcinoma of the ovary. Axial and Coronal reconstructed CT demonstrating non resectable dense confluent right subdiaphragmatic disease (arrows), diffuse thick plaque disease in the gastro-hepatic ligament (block arrow) and an deposit >2 cm in the hepato-duodenal ligament and porta hepatis (dashed arrows)

However large volume nodular or diffuse disease broadly defined as flat plaques greater than 2 cm thick are potentially non-resectable disease and should be highlighted to the surgeon [Fig. 4].

**Omental disease:**

**Fig. 5:**
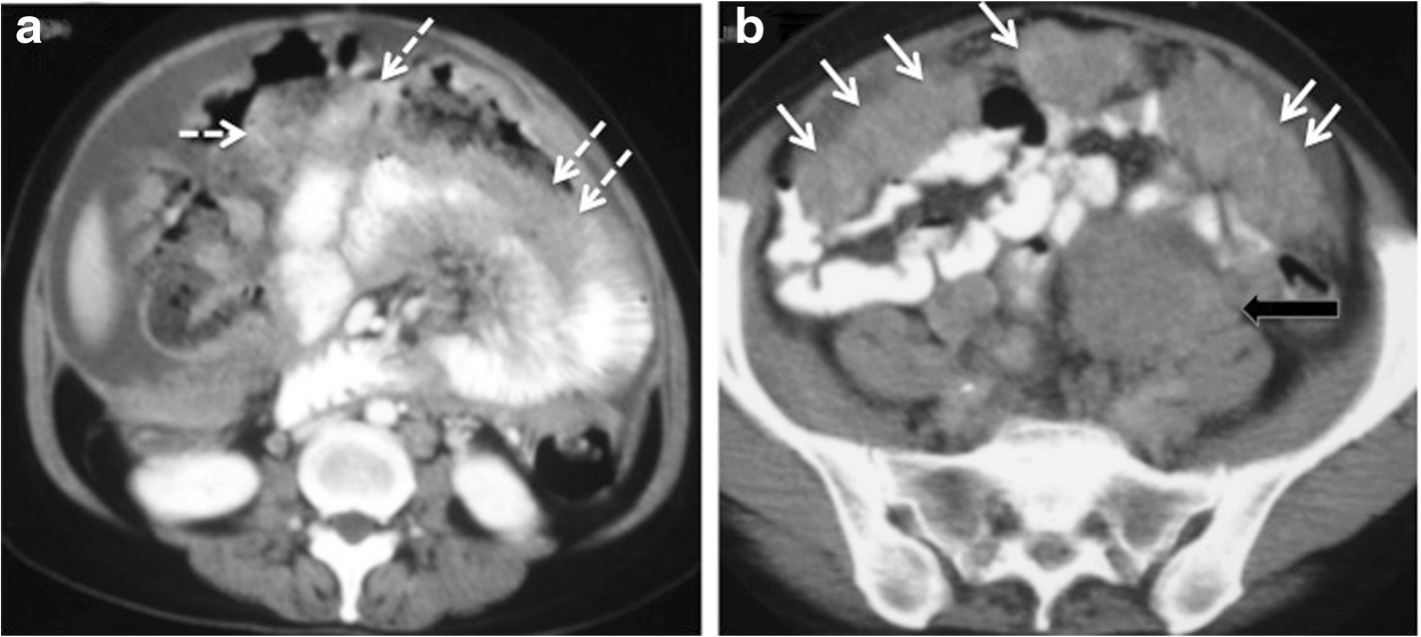
**a** and **b** 76 year old woman with non resectable stage IV undifferentiated adenocarcinoma of the ovary. The dashed arrows show extensive diffuse meso colic disease posterior to the transverse colon confluent with diffuse small bowel serosal disease. The disease results in small bowel obstruction. Solid arrows show a typical thick omental cake in the lower abdomen and a large peritoneal deposit (block arrow)

Subtle early omental disease manifests as stranding, fine reticular nodular enhancement whilst more advanced disease is plaque like and forms the classical ‘omental cake’ appearance. Resection of the omentum is part of the standard cyto-reductive surgery but involvement of the transverse meso colon, forming the supra-colic omentum will require a modified surgical approach and needs to be highlighted to the surgeon [Fig. 5].

**Upper abdominal ligaments:**

The most important upper abdominal ligaments form parts of the lesser omentum, lesser sac and perihepatic space.

The lesser omentum is made up of the horizontal hepatoduodenal ligament and vertical gastrohepatic ligament. The hepatoduodenal ligament is between porta hepatis and first part of the duodenum containing the portal vein, common bile duct, hepatic artery and lymph nodes. The gastrohepatic ligament is identified by recognizing the left gastric artery [Fig. 4]. Disease at these sites almost always complicates surgical approach, increases morbidity and requires input from the hepato-biliary surgeons. Nodular or plaque disease greater than 2 cm precludes surgical resection.

**Fig. 6:**
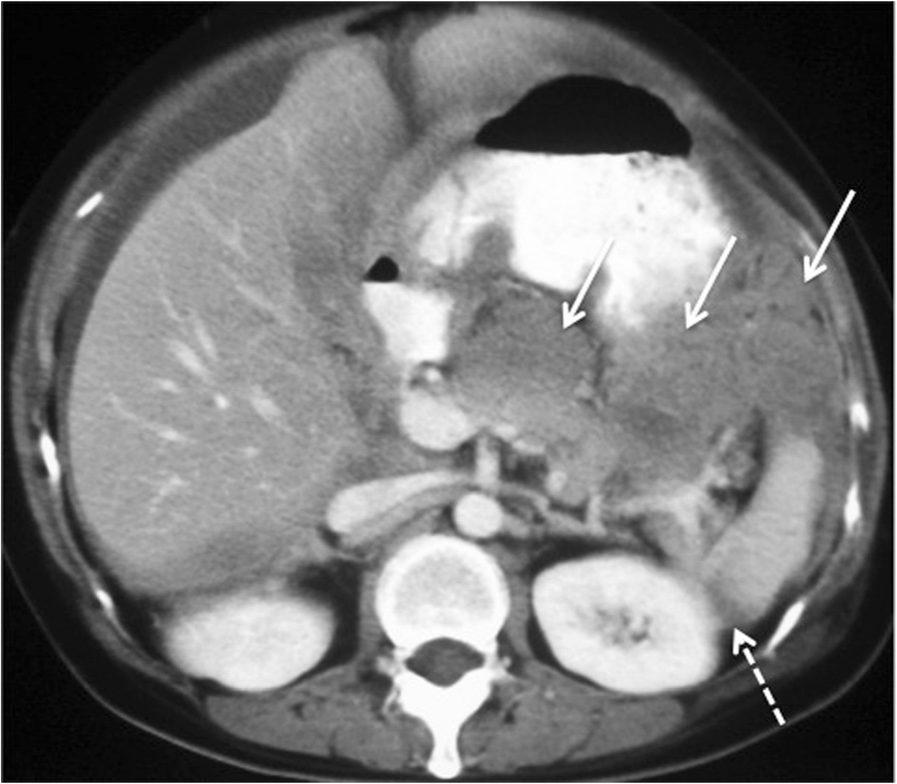
Large volume >2 cm non-resectable disease in the lesser sac (arrows) with confluent disease between the pancreas and stomach and the gastro-splenic ligament. Further disease is present in the spleno-renal ligament (dashed arrows) and ascites around the liver

The lesser sac lies between the stomach and pancreas, made up of the spleno-renal and spleno-gastric ligament connecting the greater curve of the stomach to the spleen. Involvement of these ligaments must be reported as it frequently precludes surgery [Fig. 6].

The perihepatic space refers to the falciform ligament, Morrisons pouch and the gallbladder fossa [Fig. 4]. Deposits greater than 2 cm at these sites frequently precludes surgery.

**Mesenteric disease:**

**Fig. 7:**
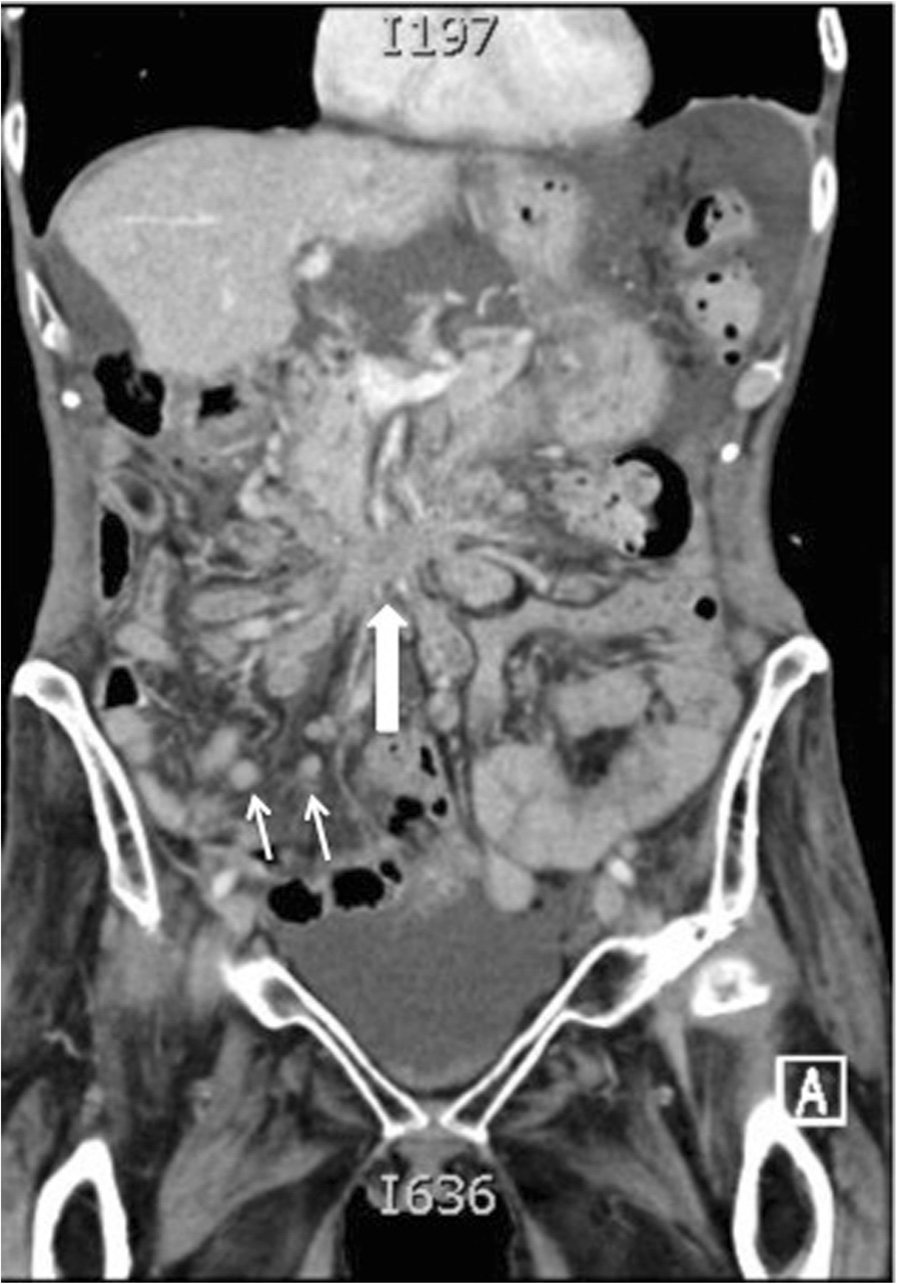
Sixty-six year old woman with Stage IV serous cystadenocarcinoma of the ovary. Dense infiltrative non-resectable disease in the mid abdominal mesentery (block arrow) with resultant cicatrization of the surrounding bowel loops. Innumerable additional nodular deposits (arrows) are scattered in the remaining small bowel mesentery which would also constitute non resectable disease

Detection and extent of mesenteric disease is particularly important to document and describe in its extent as it usually precludes surgery at most institutions. Mesenteric disease is most frequent at the right lower quadrant, in the root of the small bowel mesentery close to the terminal ileum. Early disease comprises of a misty stranded mesentery, small scattered nodules and advanced disease is plaque like with retraction and distortion of the bowel loops [Fig. 7].

### Lymphadenopathy

**Fig. 8:**
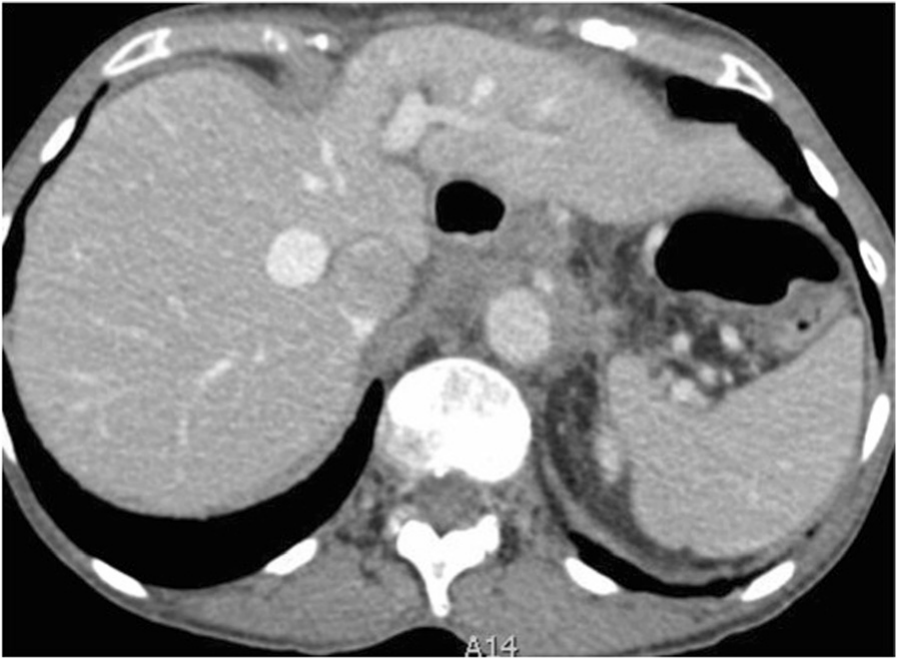
Seventy-three year old woman with serous cystadenocarcinoma. Extensive diffuse non resectable retroperitoneal nodal metastases encasing the aorta, root of coeliac artery and invading the diaphragm (arrows). Enlarged right para-cardiac node (dashed arrow) indicating stage IV disease

Pre-operative detection of lymphadenopathy allows surgical modification to include retroperitoneal node dissection laproscopically as a two part surgical procedure or as an extended single surgical procedure. At present the nodal short axis diameter of 1 cm is used to suggest malignant lymphadenopathy. Diffuse retroperitoneal nodal disease in the supra-renal distribution should be highlighted and may preclude surgery. Detection of para-cardiac nodes ≥1 cm may preclude to surgery and confers a poor prognosis [5, 10] [Fig. 8].

### Visceral metastases

**Fig. 9:**
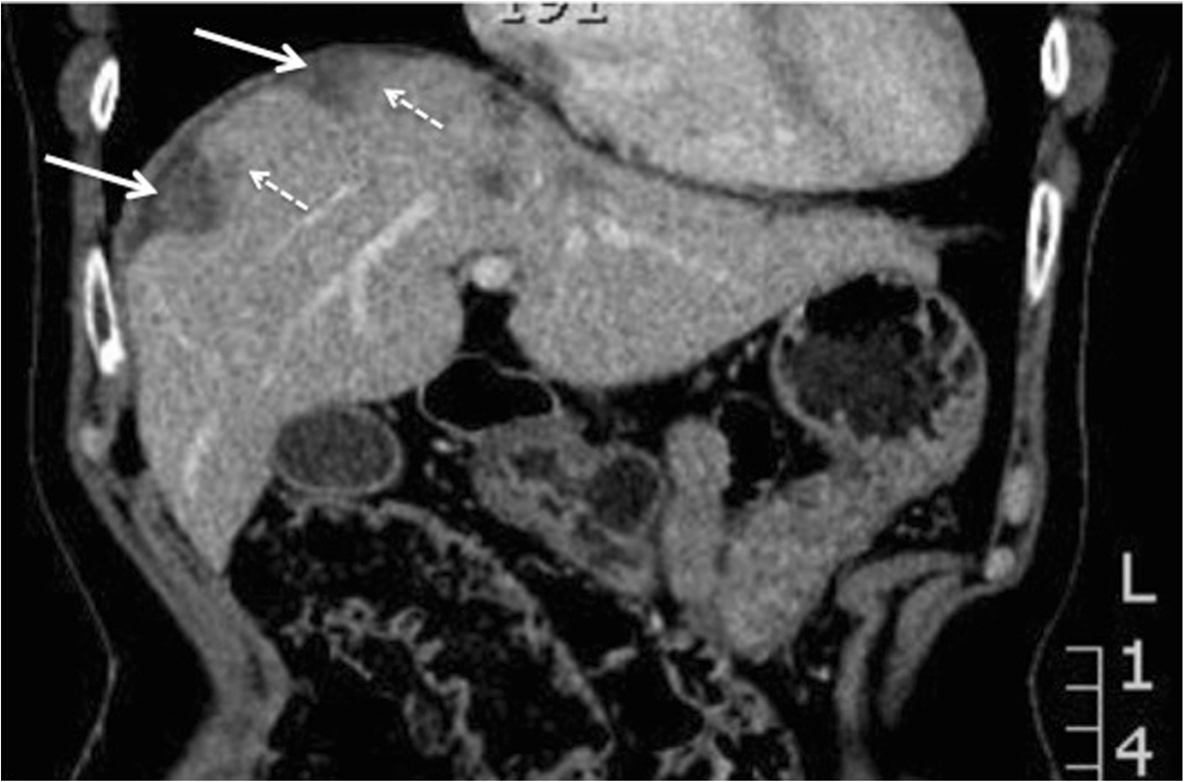
Fifty-five year old woman with endometriod carcinoma of the ovary. Hepatic subcapsular deposits (arrows) with invasion of underlying liver parenchyma (dashed arrows). The invasion requires hepato-biliary surgical assistance as segmentectomy or lobectomy may be required in patients being considered for optimal resection

True haematogenous parenchymal metastases are extremely rare at presentation. Invasive serosal surface implants are the most frequent mode of parenchymal involvement of liver and spleen and these may invade the underlying liver parenchyma. Surface hepatic implants can be easily strummed at surgery whilst deeply invasive implants require partial segmental or lobar hepatectomy and the extent of surgical resection requires pre-operative liaison with hepatobiliary surgeons. Implants in Morrisons pouch extending to hepatic porta and IVC are said to increase the risk of sub optimal resection and intraoperative bleeding [Fig. 9].

The distinction between surface and invasive implants is less critical for the spleen as a splenectomy is more easily performed. The presence of splenic hilar disease increases the likelihood of a splenectomy and patients will require pre operative vaccination and antibiotic prophylaxis.

### Bowel metastases

Involvement of bowel is frequent in advanced disease and occurs by direct invasion from the pelvic carcinomas, along mesenteric fascial planes, through lymphatic channels, by intraperitoneal seeding of tumour or by embolic haematogenous spread.

**Fig. 10:**
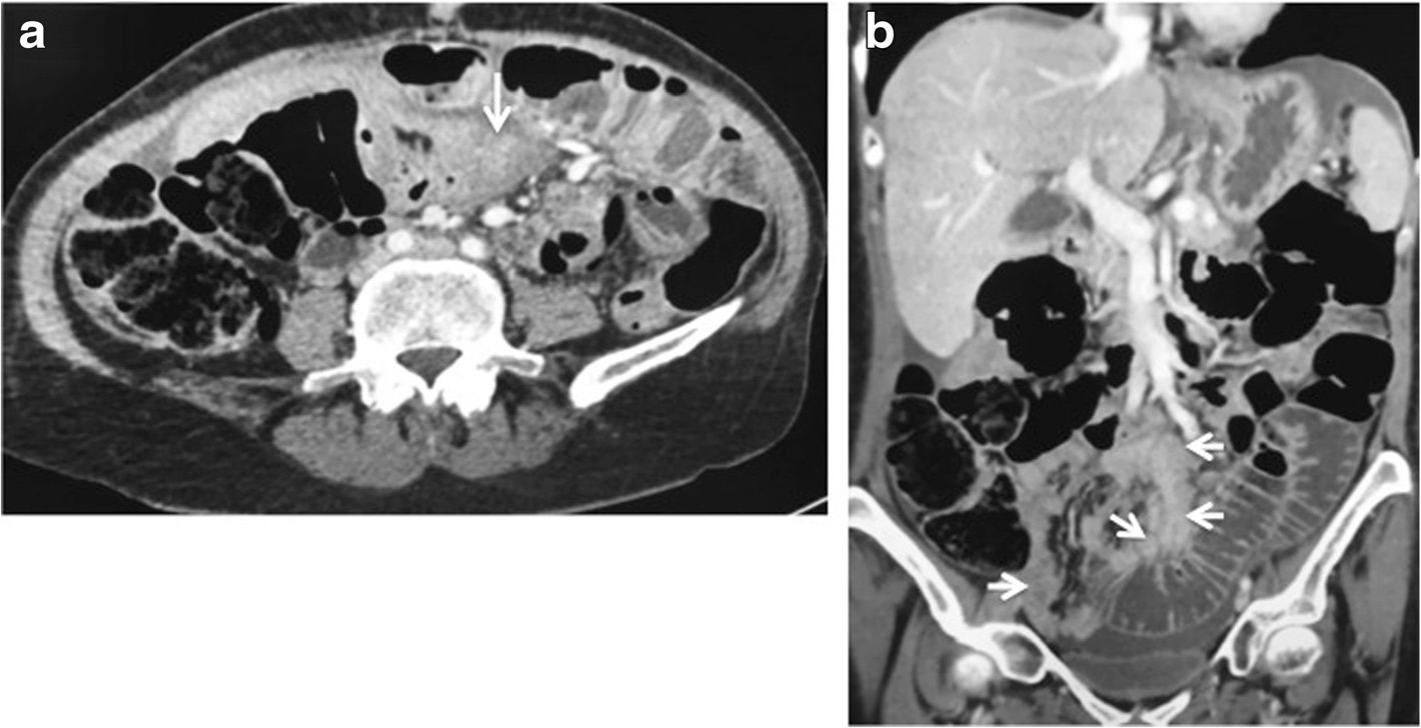
58 year old woman with stage IIIC undifferentiated carcinoma of the ovary. Axial (**a**) and coronal CT (**b**) shows extensive small bowel mesenteric disease (arrows) invading small bowel serosa and wall with resultant small bowel obstruction. Resection of this degree of mesenteric invasion would require sacrificing the entire small bowel

It is the most common cause of ovarian cancer–associated morbidity. Bowel involvement may be nodular or plaque like lesions along serosal and wall surfaces and when bowel wall invasion is also present, obstruction is a common complication. In patients with bowel obstruction, evaluation of obstructing disease provides important information to assess the role of surgery. The presence of multiple obstructing lesions, diffuse bowel plaque disease and extensive invasion of the bowel mesentery are features precluding surgery. High obstruction of small bowel is also a poor prognostic feature as decompression requires high output stomas which are associated to metabolic complications [Fig. 10].

#### B: Reporting template

Structured reporting is strongly recommended and should include features of abdominal and pelvic disease in detail. This structure can be presented as suggested in Table 1.

**Table 1:** Suggested structured presentation of the radiological report

Sites of Resectable Disease	
Pelvis	
Abdomen	
Retroperitoneum	
Chest (if included)	
Potentially non resectable disease	
Pelvis	
Abdomen	
Retroperitoneum	
Chest (if included)	
Non resectable disease	
Pelvis	
Abdomen	
Retroperitoneum	
Chest (if included)	
Disease Complications	
Stage of ovarian carcinoma	
Radiological FIGO stage	
Other significant findings	
eg. AAA, unexpected non ovarian malignancy	

The CT report should include:

1. The categories of disease including details of pelvic disease, other sites of resectable disease, and alerting clinicians to sites of possibly non resectable and non resectable disease.
2. Sites of disease that may not be visible at laparoscopy. Intraluminal tumour deposits in bowel and bladder, small parenchymal metastases to the liver and spleen and pleural metastases may not be appreciated at laparoscopy.
3. Complications of ovarian cancer, which include bowel obstruction, hydronephrosis and venous thrombosis in the pelvis, IVC or pulmonary emboli.
4. The radiological FIGO stage of ovarian cancer. Table 2 summarises the new FIGO 2014 staging of ovarian cancer.

**Table 2:** FIGO staging of ovarian cancer 2014 [11]

Stage I: Tumour confined to ovaries	1A: Tumor limited to 1 ovary, capsule intact, no tumor on surface, negative washings.
	1B: Tumor involves both ovaries otherwise like IA.
	1C: Tumor limited to 1 or both ovaries
	IC1: Surgical spill
	IC2: Capsule rupture before surgery or tumor on ovarian surface.
	IC3: Malignant cells in the ascites or peritoneal washings.
Stage II: Tumor involves 1 or both ovaries with pelvic extension (below the pelvic brim) or primary peritoneal cancer	11A: Extension and/or implant on uterus and/or Fallopian tubes
	IIB: Extension to other pelvic intraperitoneal tissues
Stage III: Tumor involves 1 or both ovaries with cytologically or histologically confirmed spread to the peritoneum outside the pelvis and/or metastasis to the retroperitoneal lymph nodes	IIIA: Positive retroperitoneal lymph nodes and /or microscopic metastasis beyond the pelvis
	IIIA1: Positive retroperitoneal lymph nodes only
	IIIA1(i) Metastasis ≤ 10 mm
	IIIA1(ii) Metastasis > 10 mm
	IIIA2: Microscopic, extrapelvic (above the brim) peritoneal involvement ± positive retroperitoneal lymph nodes
	IIIB: Macroscopic, extrapelvic, peritoneal metastasis ≤ 2 cm ± positive retroperitoneal lymph nodes. Includes extension to capsule of liver/spleen.
	IIIC: Macroscopic, extrapelvic, peritoneal metastasis > 2 cm ± positive retroperitoneal lymph nodes. Includes extension to capsule of liver/spleen.
Stage IV: Distant metastasis excluding peritoneal metastasis	IVA: Pleural effusion with positive cytology
	IVB: Hepatic and/or splenic parenchymal metastasis, metastasis to extra- abdominal organs (including inguinal lymph nodes and lymph nodes outside of the abdominal cavity)
5. Suitable modality and sites of disease for image guided biopsy in patients likely to undergo primary chemotherapy

## Conclusion and key points

1. The majority of ovarian cancer patients present with stage III and IV disease. The optimal management is broadly accepted as optimal cyto-reductive surgery, which may need to be supplemented by chemotherapy.
2. The criteria for assessing surgical resectability are not universally accepted. Each institution will set its own criteria depending on local surgical and oncological expertise and the patients’ clinical status.
3. However a systematic review of the staging CT contributes significantly to decision-making and identifies resectable and non-resectable sites of disease, which alter the patient’s management at presentation.
4. A structured presentation of these findings not only provides the surgeons a clear road map for surgery but allows the pre-operative input of other subspecialty surgeons who may be required to achieve complete (R0) or near complete resection (R1) of disease.
5. For predicting non-resectability, CT plays a critically important role in identifying lesions >2 cm at the root of the mesentery, gastro-splenic ligament, lesser sac, porta hepatis, falciform ligament, para-cardiac nodes and lung parenchyma, and also in detecting high retroperitoneal lymphadenopathy, presacral extraperitoneal disease, and pelvic sidewall invasion.

As progress continues in surgical technique and according to available expertise, the boundaries between resectable and non resectable disease will continue to evolve. However if these sites of disease are clearly stated in the CT report in a template format, clinical discussion will correctly triage the patients into appropriate treatment categories.

## Abbreviations

CT, computed tomography; FIGO, International Federation of Gynecology and Obstetrics; R0, complete surgical resection; R1, residual disease less than 1 cm.
